# Study on Large‐Scale Brain Network Abnormalities in Patients With Beta‐Thalassemia

**DOI:** 10.1002/brb3.70614

**Published:** 2025-06-17

**Authors:** Mingrui Yang, Guowei Chen, Peng Peng, Cheng Tang, Chaotian Luo, Fei Peng, Rong Kong, Chunxia Zhu, Jiatong Liang

**Affiliations:** ^1^ Department of Radiology The First Affiliated Hospital of Guangxi Medical University Nanning China; ^2^ Binzhou Medical University Hospital Binzhou China; ^3^ NHC Key Laboratory of Thalassemia Medicine Nanning China

**Keywords:** functional connectivity, functional network connectivity, independent component analysis, resting‐state functional network, β‐thalassemia

## Abstract

**Background:**

Beta‐thalassemia major (β‐TM) is a hereditary blood disorder characterized by chronic anemia and hypoxia, which may have profound effects on brain function. This study systematically evaluates alterations in both intra‐brain network functional connectivity (FC) and inter‐network functional connectivity (FNC) in β‐TM patients using resting‐state functional magnetic resonance imaging (rs‐fMRI) and independent component analysis (ICA), aiming to uncover the potential mechanisms underlying their neurofunctional impairments.

**Methods:**

This study included 72 β‐TM patients and 50 age‐ and gender‐matched healthy controls (HC). rs‐fMRI was used to collect brain functional data, and ICA was applied to extract 14 resting‐state functional networks (RSNs). Differences in FC within networks and FNC between the two groups were further compared to investigate the brain network abnormalities in β‐TM patients.

**Results:**

In β‐TM patients, FC within brain networks was significantly reduced in the anterior default mode network (aDMN), posterior default mode network (pDMN), left frontoparietal network (lFPN), right frontoparietal network (rFPN), ventral attention network (VAN), and executive control network (ECN). In contrast, FC was significantly increased in the dorsal sensorimotor network (dSMN) and posterior visual network (pVN). FNC analysis revealed that β‐TM patients exhibited enhanced connectivity between the lFPN and rFPN, as well as between the dorsal attention network (DAN) and VAN. However, connectivity was significantly weakened between the DAN and lFPN, ECN, auditory network (AN), and salience network (SN); as well as between the pVN and dSMN. These findings suggest impairments in cognitive control, attention allocation, and sensory integration, with specific disruptions in the SN that may contribute to the observed dysfunctions.

**Conclusion:**

Brain network abnormalities in β‐TM patients manifest as an alternating pattern of enhanced and weakened connectivity, revealing the profound impact of chronic anemia and hypoxia on cognitive, emotional, and sensory functions.

## Graphical abstract

This study reveals altered brain network connectivity in beta‐TM patients, reflecting the impact of chronic anemia and hypoxia on cognitive, emotional, and sensory functions. It identifies potential neurofunctional biomarkers and offers insights into mechanisms underlying neurocognitive dysfunctions, providing a foundation for personalized interventions and future longitudinal research.

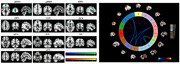

## 1 Introduction

Beta‐thalassemia major (β‐TM) is a chronic blood disorder caused by genetic mutations and is classified as a severe subtype of thalassemia (Origa 2017). It is characterized by insufficient hemoglobin (Hb) synthesis, leading to chronic anemia and iron overload due to long‐term blood transfusions (Taher and Saliba 2017). Although transfusion and iron chelation therapies have significantly extended the lifespan of β‐TM patients, persistent anemia, hypoxic conditions, and treatment‐related iron overload can have profound effects on multiple organ systems, including the central nervous system (CNS) (Manara et al. 2019). In recent years, increasing attention has been directed toward the cognitive, emotional, and behavioral abnormalities observed in β‐TM patients, as these neurofunctional impairments may significantly impact their quality of life (Bu et al. 2023). However, research on brain network connectivity changes in β‐TM patients, especially regarding functional network connectivity (FNC) under resting‐state conditions, remains limited.

Previous studies have shown that β‐TM patients often exhibit varying degrees of cognitive dysfunction, primarily affecting executive function, attention, memory, and language abilities, with these issues being especially pronounced in pediatric and adolescent patients (Elalfy et al. 2017). In addition, these patients frequently experience psychological problems such as anxiety, depression, and mood fluctuations, suggesting that these neurobehavioral manifestations may be closely linked to structural and functional abnormalities in the CNS (Raz et al. 2019). Neuroimaging studies have revealed significant changes in cerebral blood flow perfusion in β‐TM patients, indicating that chronic anemia and hypoxia may contribute to CNS damage (Manara et al. 2024). However, the changes in brain network interaction in β‐TM patients remain poorly understood, limiting the exploration of the mechanisms underlying their neurofunctional impairments.

Resting‐state functional connectivity (FC) and FNC are important indicators for assessing the interactions between brain regions (Hohenfeld et al. 2018; Hausman et al. 2020). FC primarily measures the synchronous activity between brain regions at rest, reflecting connectivity strength within local brain networks, while FNC reveals the connectivity patterns between different functional networks (Schurz et al. 2020; Deng et al. 2016). Several studies have shown that the brain consists of multiple resting‐state networks (RSNs), including the default mode network (DMN), auditory network (AN), executive control network (ECN), and visual network (VN), among others. These networks play essential roles in maintaining basic cognitive functions. For instance, the DMN is associated with self‐reflection and internal thought processes, the AN is involved in sustaining attention and integrating sensory inputs, the ECN is responsible for decision‐making and task switching in complex cognitive tasks, and the VN is closely related to the processing and integration of visual information (Smith et al. 2009; Kaiser et al. 2015; Menon 2023). Abnormal connectivity among these networks may serve as a key mechanism underlying the cognitive and emotional dysfunctions observed in β‐TM patients.

In recent years, resting‐state functional magnetic resonance imaging (rs‐fMRI) has been widely applied to the study of brain functional networks. By detecting blood oxygen level‐dependent (BOLD) signals, rs‐fMRI provides a means of dynamically observing brain network activity. When combined with independent component analysis (ICA), it is possible to extract RSNs from complex fMRI data and systematically analyze changes in FC within networks and FNC between networks (Beckmann and Smith 2004; Iraji et al. 2023). This approach provides a reliable method for investigating the potential relationships between FC and neurofunctional impairments in β‐TM patients.

Based on this, the present study aims to use rs‐fMRI and ICA to systematically assess changes in FC within brain networks and FNC between networks in β‐TM patients. The focus will be on analyzing the FC characteristics of key networks, such as the anterior default mode network (aDMN), posterior default mode network (pDMN), AN, and ECN, and exploring the potential relationship between these changes and cognitive dysfunction in patients. This study provides foundational insights that could contribute to future research on neurocognitive assessment and personalized interventions for β‐TM patients. We hypothesize that alterations in FC in β‐TM patients may be directly linked to their pathophysiological conditions, such as chronic anemia, altered blood oxygenation, and fatigue. These conditions are believed to affect brain network connectivity, which may contribute to cognitive, emotional, and sensory dysfunctions observed in β‐TM patients. However, this study does not include clinical scale analysis, so we are unable to explore direct correlations between clinical measures (such as fatigue or blood oxygenation levels) and changes in brain connectivity. Nonetheless, the observed changes in brain connectivity may still reflect underlying pathophysiological mechanisms related to chronic anemia and hypoxia.

## 2 Methods

### 2.1 Participants

A total of 125 participants were recruited for this study, comprising 75 β‐TM patients (β‐TM group) and 50 healthy controls (HC group). Age, gender, and education level were matched between the two groups. All participants underwent assessments in the same setting and provided written informed consent to participate in the study. All procedures were conducted in accordance with the Declaration of Helsinki and were approved by the Medical Ethics Committee of the First Affiliated Hospital of Guangxi Medical University, Guangxi, China.

The inclusion criteria for β‐TM patients were as follows: (1) a genetic diagnosis of β‐thalassemia major with a history of regular blood transfusions, (2) age over 15 years, and (3) successful completion of a 3T MRI scan. In addition, patients' transfusion and chelation therapy history was carefully documented, including the time of the last transfusion and the most recent chelation therapy session. These treatment details were included as covariates in the analysis to account for their potential effects on the BOLD signal. Exclusion criteria included: (1) significant MRI artifacts that could interfere with data analysis, (2) contraindications for MRI, and (3) a history of head trauma or neurosurgery. Ultimately, 72 β‐TM patients met the inclusion criteria and were included in the study.

The enrolled β‐TM patients underwent a comprehensive clinical assessment by a hematologist. All patients had a history of regular blood transfusions, which is essential for managing their condition. During the clinical assessment, the severity of anemia was classified as moderate based on standard clinical criteria. The Hb levels in the β‐TM patients were as follows: the average Hb level was 8.5 g/dL (range: 6.5–10.0 g/dL). According to the World Health Organization (WHO) criteria for anemia, an Hb level of less than 10 g/dL is classified as moderate anemia. In addition to anemia, the patients also underwent assessments for blood oxygen saturation levels, which were found to be slightly reduced, with an average oxygen saturation level of 94% (range: 92%–96%). These clinical factors, including the severity of anemia and reduced oxygen saturation, are likely to influence brain function and may contribute to changes in brain network connectivity.

Healthy controls were recruited through advertisements and community outreach, with exclusions for individuals with neurological disorders, psychiatric conditions, cardiovascular diseases, diabetes, or any chronic illnesses, as well as those with a family history of genetic disorders. The HC group was matched for age, gender, and education level with the β‐TM group. In total, 50 healthy volunteers were included as the control group.

### 2.2 Rs‐fMRI Data Acquisition

Scanning was performed using a Siemens Prisma 3T system (Siemens, Erlangen, Germany). Participants were instructed to remain still, relaxed, and with their eyes closed, while maintaining a wakeful state and refraining from thinking about specific tasks. Foam pads and head straps were used to minimize head movement. The collected data included routine T2‐weighted imaging (T2WI) for detecting structural abnormalities, such as cerebral ischemia, and for assessing overall brain morphology. High‐resolution T1‐weighted imaging (T1WI) was primarily used for assessing structural integrity and for performing image registration, ensuring accurate alignment of functional imaging data (rs‐fMRI) to a common anatomical space.

Resting‐state fMRI data were acquired using a gradient echo planar imaging (EPI) sequence with the following parameters: repetition time (TR) = 2000 ms, echo time (TE) = 30 ms, flip angle = 90°, field of view (FOV) = 224 mm × 224 mm, matrix size = 64 × 64, slice thickness = 3.5 mm, slice gap = 0.5 mm, covering 33 axial slices to encompass the entire brain. Each participant underwent an 8‐min rs‐fMRI scan, resulting in 240 time points.

The high‐resolution T1WI parameters were as follows: TR/TE = 1900/2.26 ms, FOV = 215 × 230 mm, matrix size = 240 × 256, and 176 sagittal slices with a thickness of 1.0 mm. For T2WI, the scanning parameters were: TR/TE = 5100/117 ms, FOV = 240 × 240 mm, matrix size = 416 × 416, with three excitations, an echo train length of 11, and 22 axial slices with a thickness of 6.5 mm.

Both T1WI and T2WI scans were conducted on all β‐TM patients and HC to ensure consistency in the assessment of brain structure across both groups. This approach allowed for a comprehensive comparison of brain structure and FC, providing a reliable basis for our analysis.

### 2.3 Data Preprocessing

All preprocessing was performed using the RESTPlus toolbox (http://restfmri.net/forum/rest‐plus), which is based on Statistical Parametric Mapping (SPM12) (http://www.fil.ion.ucl.ac.uk) and implemented in MATLAB 2022b (MathWorks, Natick, MA, USA). The preprocessing steps for the raw data were as follows: (1) The first 10 time points were discarded to allow the signal to stabilize and to exclude potential artifacts from the initial scan. (2) Head motion correction was performed by aligning the images from each time point to the image from the first time point, thereby correcting for motion‐induced image distortions. Participants with head movement parameters exceeding 3 mm for translation or 3.0° for rotation were excluded from subsequent analysis. (3) Spatial normalization was conducted to align all participants' images to the standard Montreal Neurological Institute (MNI) space to facilitate group comparisons. (4) Spatial smoothing was applied using a Gaussian kernel with a full‐width at half‐maximum (FWHM) of 6 mm to improve the signal‐to‐noise ratio of the images. (5) A temporal bandpass filter (0.01–0.1 Hz) was applied to the fMRI time series to remove low‐frequency drift and high‐frequency physiological noise such as cardiac and respiratory signals. This frequency range was selected to match the typical characteristics of spontaneous BOLD signal fluctuations in RSNs, as well as to avoid contamination from non‐neuronal sources. The bandpass‐filtered data were subsequently used as input for ICA.

### 2.4 Independent Component Analysis

Group ICA was conducted using the GIFT toolbox (http://icatb.sourceforge.net/, version 3.0b) on temporally filtered data (0.01–0.1 Hz), which was obtained from the preprocessing stage. This ensured that the input to ICA contained only low‐frequency BOLD signal fluctuations relevant for RSN identification and excluded higher‐frequency physiological noise. The ICA decomposition produced a set of spatially independent components (ICs), each representing a putative RSN. The decomposition process followed several steps. Initially, the software estimated 63 ICs and computed the spatial correlation of the BOLD signals using the minimum description length criterion. After ICA decomposition, data from individual participants were reviewed to identify and remove any ICs that contained artifacts, such as head motion, vascular contributions, or other non‐neuronal signals. These components were marked as ‘bad’ and excluded from the analysis. On average, approximately 11% of the ICs were removed from each participant's data due to these criteria.

To further refine the data, principal component analysis (PCA) was used to reduce the dimensionality of the combined dataset. PCA was employed primarily to normalize the data across subjects, ensuring that FC comparisons between participants were standardized. While ICA reduced the data into ICs, PCA was applied to standardize the variance across subjects, which allowed for more accurate cross‐subject comparisons by normalizing inter‐subject variability.

At the group level, ICA was applied to back‐reconstruct the ICs for each participant. To identify brain networks, the following selection criteria were applied: (1) the spatial peaks of each network were located in the gray matter, (2) the distribution of the network did not show significant vascular artifacts, assessed using a vascular template derived from known brain regions associated with large blood vessels, such as the middle cerebral artery, and spatial correlation with vascular regions identified through ICA, and (3) the network's signal was within the low‐frequency range (0.01–0.1 Hz).

To obtain subject‐specific spatial maps and time series for each of the 14 networks, we performed a dual regression analysis. This allowed us to extract individual patterns of brain activity for each participant, based on the group‐level ICA decomposition. Dual regression is useful as it takes group‐level network components and regresses them back onto individual data to obtain subject‐specific spatial maps and associated time series, providing a more personalized measure of connectivity. In this study, we chose to use ICA rather than relying on conventional RSN parcellation methods. ICA is a data‐driven technique that does not assume any prior information about the spatial distribution of networks. It allows us to identify ICs that represent meaningful neural activity across participants without being restricted to pre‐defined network templates. Although known RSN patterns exist, they are based on fixed templates and may not capture the full complexity or variability of brain networks. ICA, by contrast, offers a more flexible and individualized approach to network identification, particularly when exploring complex patterns in populations like β‐TM patients.

In this study, 14 RSNs were identified, including the aDMN, pDMN, dorsal sensorimotor network (dSMN), ventral sensorimotor network (vSMN), left frontoparietal network (lFPN), right frontoparietal network (rFPN), dorsal visual network (DVN), ventral attention network (VAN), ECN, medial visual network (mVN), lateral visual network (lVN), posterior visual network (pVN), AN, and salience network (SN).

### 2.5 Within‐Network FC Analysis

Spatial maps for each RSN were analyzed using a one‐sample t‐test in SPM12, with statistical significance set at *p* < 0.001, corrected for family‐wise error (FWE). This *p*‐value threshold was applied at the voxel level to identify individual voxels that exhibit statistically significant activity. Following this, clusters of significant voxels were identified using a more lenient threshold of *p* < 0.05 at the cluster level, to identify meaningful brain regions or clusters. The two‐stage thresholding approach was used to control for both Type I errors at the voxel level and false positives at the cluster level. A two‐sample *t*‐test was then performed to compare the differences in FC within the RSNs between the two groups. The Gaussian random field (GRF) method was applied for multiple comparison correction, and age, gender, transfusion, and chelation therapy history were regressed using SPM12. These covariates were chosen to control for potential confounding factors that could influence FC. While these basic demographic variables were controlled for, future studies could benefit from incorporating other clinical covariates such as Hb levels, blood oxygen saturation, or psychological measures (e.g., mood and anxiety scores) to explore their potential associations with brain network changes in β‐TM patients. Group comparisons were masked to include only relevant voxels within each RSN (two‐tailed, voxel‐level *p* < 0.001, FWE‐corrected, cluster‐level *p* < 0.01).

### 2.6 Between‐Network FC Analysis

The temporal relationships between RSNs were assessed using the FNC method implemented in SPM12. Specifically, time series for each of the 14 networks were extracted using ICA. The correlations between the time series of different networks were then calculated to assess the interaction between networks over time. A two‐sample *t*‐test was used to compare the differences in FNC between the two groups, with statistical significance set at *p* < 0.001, corrected for FWE.

## 3 Results

### 3.1 Demographic Information

A total of 122 participants were included in this study. The healthy control group consisted of 50 individuals (25 male, 25 female), with an age range of 16–44 years and a mean age of 29.96 ± 6.54 years. The average years of education was 15.76 ± 2.83 years. The β‐thalassemia major (β‐TM) group included 72 participants (36 male, 36 female), with an age range of 16–57 years and a mean age of 29.69 ± 9.99 years. The average years of education was 15.58 ± 2.90 years. The average time (±SD) since the last transfusion was 25.4 ± 7.2 days, and the average time since the last chelation therapy was 30.6 ± 8.5 days. No significant differences were observed between the two groups in terms of gender, age, or years of education, indicating that the groups were comparable (Table 1).

**TABLE 1:** Demographic and clinical characteristics of β‐TM and HC groups.

Characteristic	β‐TM (*N* = 72)	HC (*N* = 50)	*p* value
Sex (Male/Female)	36/36	25/25	N/A
Age (Years)	29.69 ± 9.99	29.96 ± 6.54	0.360
Education (Years)	15.58 ± 2.90	15.76 ± 2.83	0.629
Last transfusion (days before scan)	25.4 ± 7.2	N/A	N/A
Last chelation therapy (days before scan)	30.6 ± 8.5	N/A	N/A

*Note*: Continuous variables are presented as the means ± standard deviations.

Abbreviations: β‐TM, β‐Thalassemia Major; HC, healthy control.

### 3.2 Spatial Patterns of RSNs in Each Group

**FIGURE 1:**
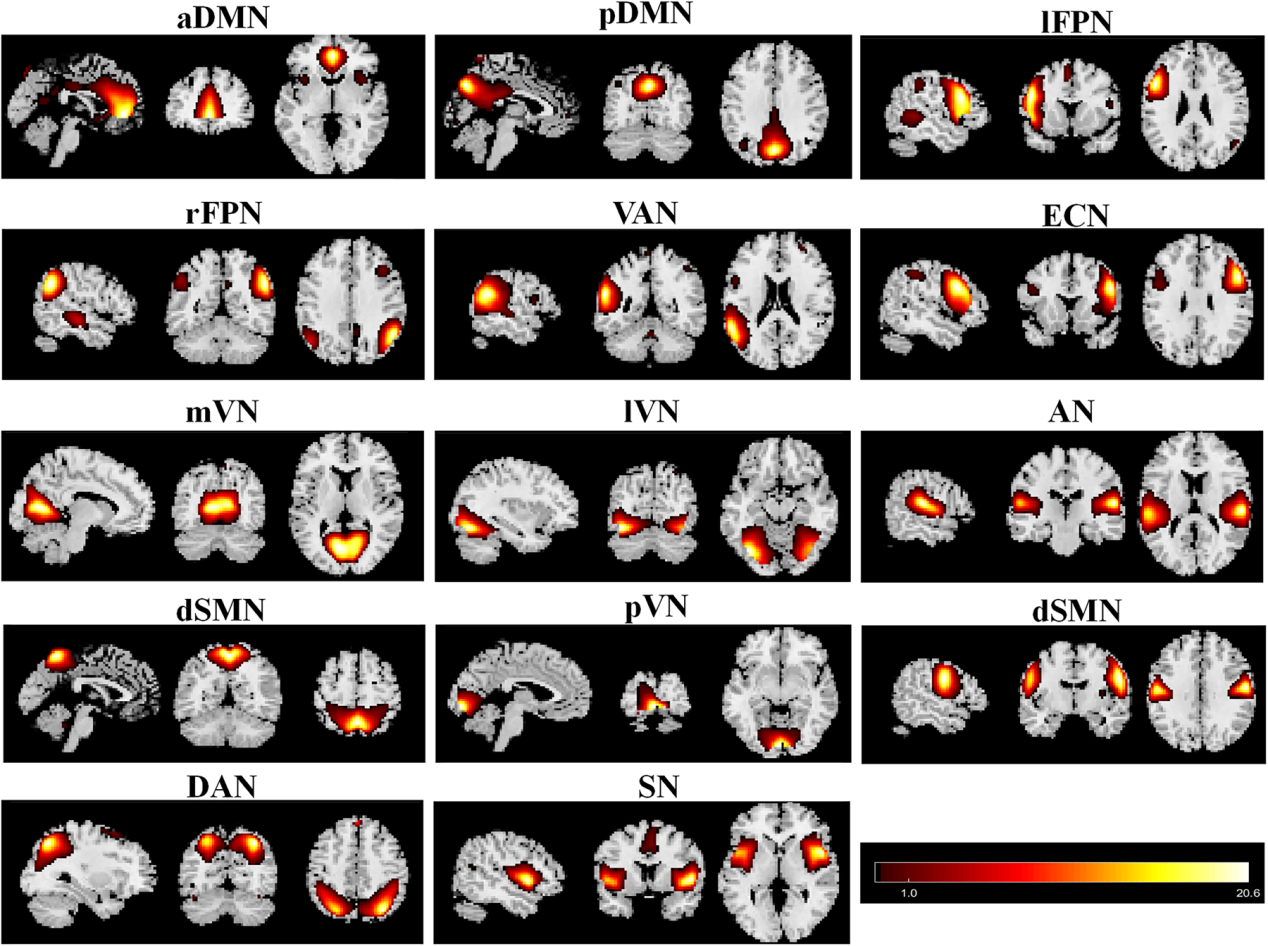
Spatial maps of 15 selected independent components. Abbreviations: aDMN, anterior default mode network; AN, auditory network; dSMN, dorsal sensorimotor network; DVN, dorsal visual network; ECN, executive control network; lFPN, left frontoparietal network; lVN, lateral visual network; mVN, medial visual network; pDMN, posterior default mode network; pVN, posterior visual network; rFPN, right frontoparietal network; SN, salience network; VAN, ventral attention network; vSMN, ventral sensorimotor network.

As shown in Figure 1, the typical spatial patterns of each RSN in the β‐TM and HC groups were identified. The 14 components corresponded to the following RSNs: aDMN, pDMN, dSMN, vSMN, lFPN, rFPN, DAN, VAN, ECN, mVN, lVN, pVN, AN, and SN (Figure 1).

### 3.3 RSN Changes in the β‐Thalassemia Major Group

**FIGURE 2:**
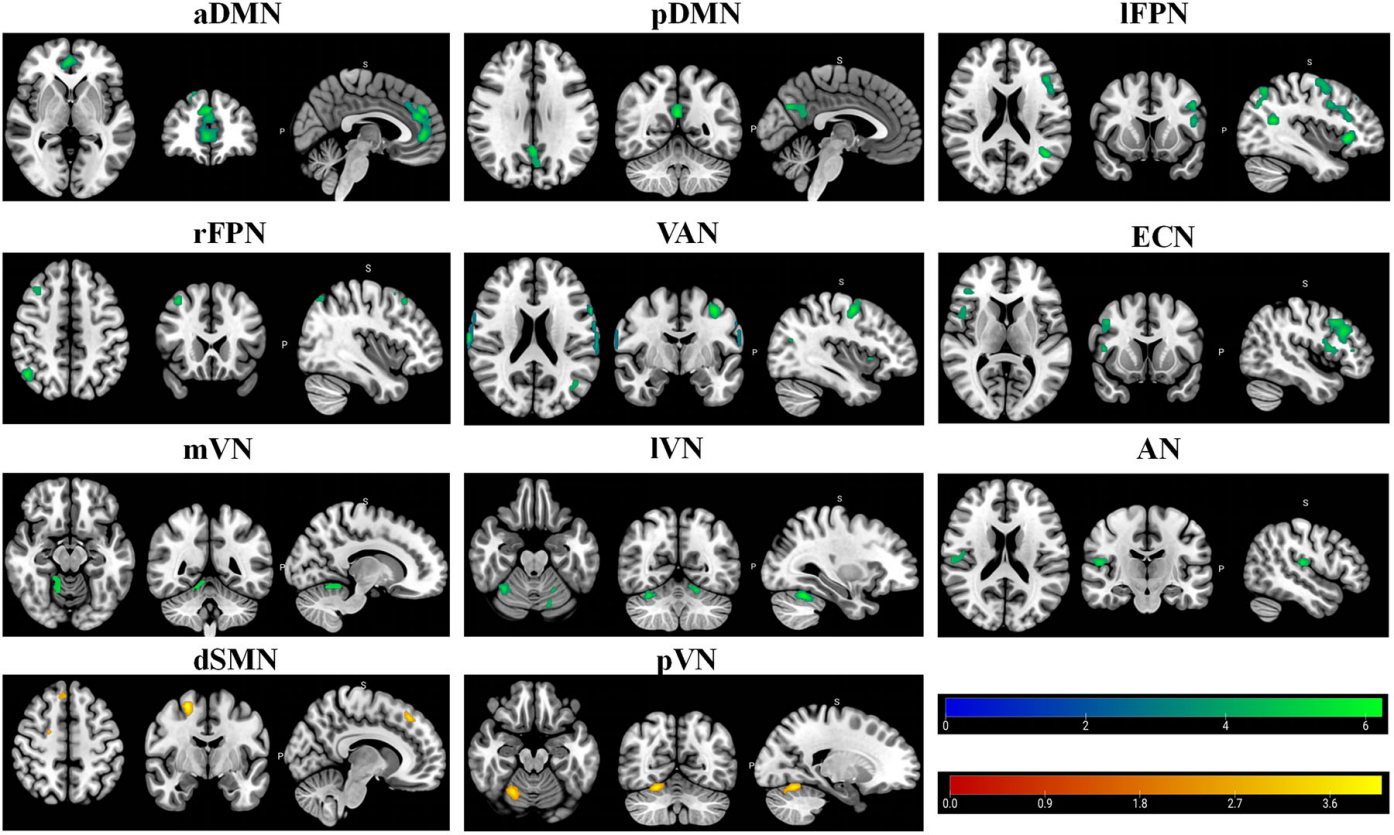
Brain regions with significant differences of intrinsic intranetwork functional connectivity between β‐TM patients and HC. All clusters survived correction for multiple comparisons with a significance threshold of a voxel‐wise value of *p* < 0.001 and a family‐wise error‐corrected *p* < 0.01 at cluster level. Warm colors (positive values) represent increased intrinsic functional connectivity, cooler colors (negative values) decreased intrinsic functional connectivity, in β‐TM patients compared to HC. Abbreviations: aDMN, anterior default mode network; AN, auditory network; dSMN, dorsal sensorimotor network; ECN, executive control network; HC, healthy control; lFPN, left frontoparietal network; lVN, lateral visual network; mVN, medial visual network; pDMN, posterior default mode network; pVN, posterior visual network; rFPN, right frontoparietal network; VAN, ventral attention network; β‐TM, β‐thalassemia major.

Compared to the HC group, β‐TM patients showed a significant increase in FC within the dSMN (right postcentral gyrus) and the pVN (left cuneus). In contrast, β‐TM patients exhibited significantly reduced FC within several networks, including the aDMN (right anterior cingulate gyrus, right superior medial frontal gyrus), pDMN (left precuneus), lFPN (left inferior frontal gyrus triangular part, left angular gyrus), rFPN (right angular gyrus), vAN (right precentral gyrus, left precentral gyrus), ECN (right inferior frontal gyrus triangular part), mVN (right cerebellar lobules IV and V), lVN (right cerebellar lobule VI, left cerebellar lobule VI), and AN (right central sulcus) (Table 2 and Figure 2).

**TABLE 2:** Intranetwork FCs of RSNs in the β‐TM group.

Condition	RSN	Brain regions	Peak T‐scores	MNI coordinates (x, y, z)	Cluster size (Voxels)
HC > β‐TM	aDMN	Right anterior cingulum	6.3920	0, 48, 6	230
		Right superior medial Frontal gyrus	5.1746	3, 48, 30	72
HC > β‐TM	pDMN	Left precuneus	4.9271	0, −54, 24	91
HC > β‐TM	lFPN	Left inferior frontal triangular part	5.3104	−45, 9, 36	175
		Left angular gyrus	4.4309	−39, −66, 51	61
HC > β‐TM	rFPN	Right angular gyrus	4.9567	45, −66, 51	49
HC > β‐TM	VAN	Right precentral gyrus	6.9774	69, −15, 21	135
		Left precentral gyrus	5.0946	−36, −3, 54	58
HC > β‐TM	ECN	Right inferior frontal triangular part	5.4215	42, 24, 30	182
HC > β‐TM	mVN	Right cerebellum lobules 4 and 5	3.9870	18, −42, −18	30
HC > β‐TM	lVN	Right cerebellum lobule 6	5.2473	30, −54, −24	56
		Left cerebellum lobule 6	4.7370	−15, −63, −15	54
HC > β‐TM	AN	Right rolandic operculum	4.8470	51, −21, 15	48
HC < β‐TM	dSMN	Right postcentral gyrus	4.1741	18, −33, 54	25
HC < β‐TM	pVN	Left calcarine sulcus	5.5694	6, −87, −6	76

*Note*: The statistical threshold was set at the voxel level with *p* < 0.001 for multiple comparisons using Gaussian random field theory (voxel‐level *p* < 0.001, FWE correction, cluster‐level *p* < 0.01). The *t*‐score represents the statistical value of peak voxels showing differences in FC between the two groups.

Abbreviations: aDMN, anterior default mode network; AN, auditory network; dSMN, dorsal sensorimotor network; ECN, executive control network; FC, functional connectivity; HC, healthy control; lFPN, left frontoparietal network; lVN, lateral Visual Network; mVN, medial visual network; pDMN, posterior default mode network; pVN, posterior visual network.; rFPN, right frontoparietal network; RSNs, resting state networks; VAN, ventral attention network; β‐TM, β‐thalassemia major.

### 3.4 FNC Analysis in the β‐Thalassemia Major Group

**FIGURE 3:**
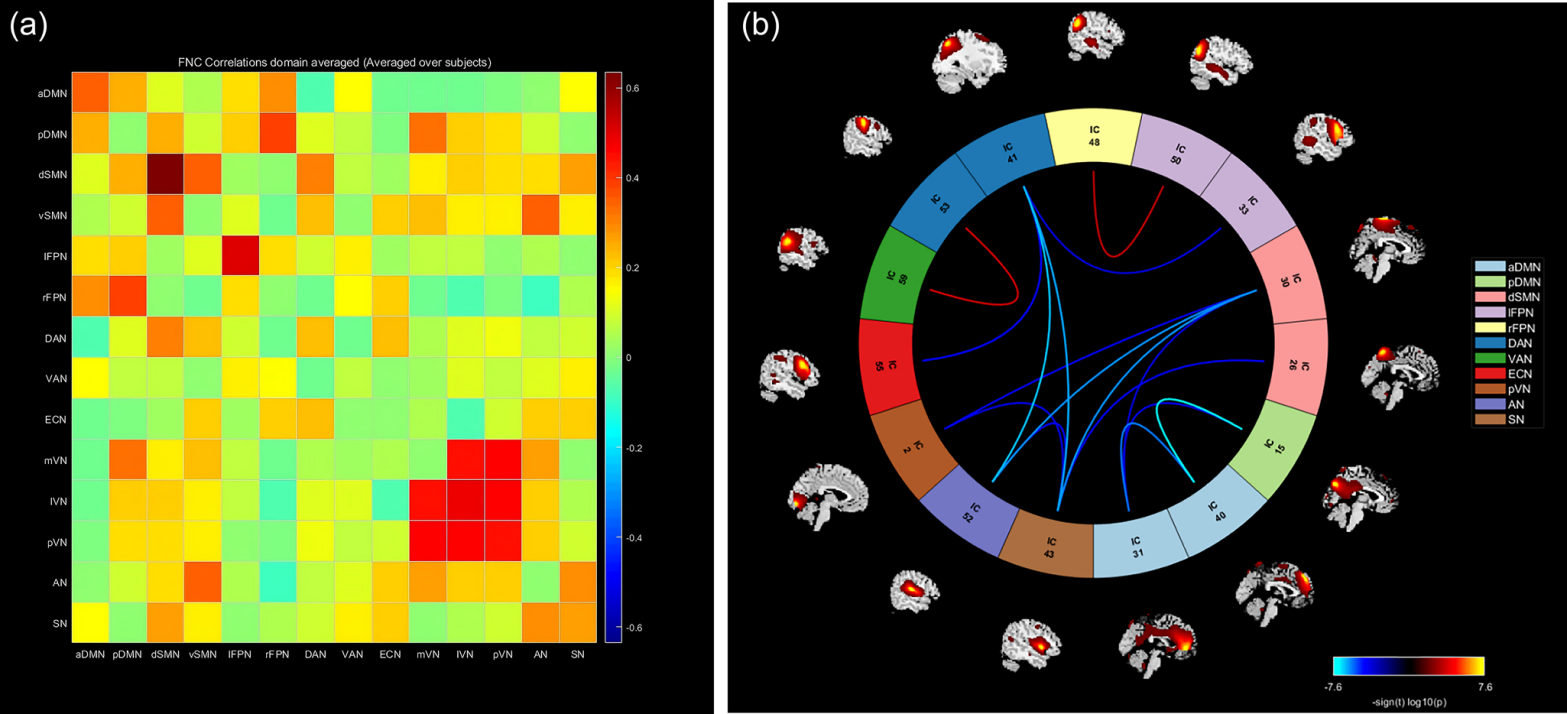
(a) Internetwork functional connectivity matrix. Pairwise correlations between resting‐state functional networks were averaged across participants. (b) Between‐group differences of inter‐network functional connectivity between β‐TM patients and HC. Warmer colors represent increased internetwork FNC, cooler colors decreased internetwork FNC in β‐thalassemia compared to healthy controls. Abbreviations: aDMN, anterior default mode network; AN, auditory network; dSMN, dorsal sensorimotor network; DVN, dorsal visual network; ECN, executive control network; lFPN, left frontoparietal network; lVN, lateral visual network; mVN, medial visual network; pDMN, posterior default mode network; pVN, posterior visual network; rFPN, right frontoparietal network; SN, salience network; VAN, ventral attention network; vSMN, ventral sensorimotor network.

Compared to the HC group, β‐TM patients exhibited a significant increase in FNC between the lFPN and rFPN, as well as between the DAN and VAN. In contrast, β‐TM patients showed significantly reduced FNC between several networks, including the DAN and lFPN, ECN, AN, and SN; the pVN and dSMN, as well as the SN; the AN and dSMN, and the SN; the SN and dSMN; and between the aDMN and pDMN, as well as the dSMN (Figure 3).

## 4 Discussion

This study is the first to systematically explore resting‐state FC and FNC in β‐TM patients, focusing on 14 distinct brain networks. The results show significant alterations in FC and FNC, which may be related to the effects of chronic anemia and hypoxia on brain function. Our findings indicate enhanced connectivity in the dSMN and pVN networks and reduced connectivity in the DMN, ECN, FPN, and VAN. These alterations are consistent with the neurofunctional impact of long‐term hypoxia, which may be adaptive at first but could lead to cognitive decline over time.

Increased connectivity in the sensory‐motor networks (dSMN, pVN) might reflect compensatory mechanisms due to chronic anemia and hypoxia (Wang et al. 2021). These networks are likely being strengthened to maintain sensory and motor functions despite the reduced efficiency of higher cognitive networks (Bonkowsky and Son 2018). However, the prolonged reliance on these networks may limit the brain's flexibility, especially in tasks requiring executive function and emotional regulation, which could lead to cognitive impairments (Chand et al. 2017). Notably, FC reductions in the DMN, ECN, FPN, and VAN networks suggest that β‐TM patients may experience deficits in higher‐order cognitive functions, including attention, memory, and executive control (Buckner and DiNicola 2019). These reductions are consistent with previous research showing that chronic hypoxia impairs brain regions responsible for attention, memory, and emotional regulation (Zhao et al. 2022; Nee 2021). Furthermore, the observed deficits in the DMN and FPN are linked to impairments in self‐reflection, memory consolidation, and task‐switching ability (Feng et al. 2019; Brissenden and Somers 2019).

Changes in FNC, particularly the increased connectivity between the lFPN and rFPN, suggest that the brain may enhance interhemispheric coordination to optimize cognitive resource allocation in response to chronic anemia and hypoxia (Lin et al. 2012). This enhanced coordination could be a result of β‐TM patients’ attempts to optimize brain resource allocation in response to increased cognitive load due to chronic anemia and hypoxia. The enhancement of FNC is not limited to interaction within the FPN. The increased connectivity between the DAN and VAN further reflects the brain's adaptive mechanism in β‐TM patients. The DAN is primarily responsible for regulating attention to external visual stimuli, while the VAN plays a critical role in detecting environmental changes (Zhao et al. 2022). This enhancement may reflect the brain's effort to improve sensory processing and responsiveness to visual and environmental changes through stronger interaction between attention networks (Suo et al. 2021).

However, despite some improvements in connectivity, the reduced FNC between key networks such as the DAN and FPN indicates deficits in cross‐modal integration and dynamic environmental adaptation. This reduction in connectivity may indicate diminished task execution efficiency in dynamic environments (Hu et al. 2023). These findings suggest that β‐TM patients may struggle to adapt to complex environments, particularly in tasks requiring rapid attention shifts and sensory integration (Steiner et al. 2022). The weakened FNC between the AN and SN, as well as the dSMN, suggests deficits in emotional regulation, sensory integration, and motor coordination in β‐TM patients (Zhou et al. 2023). The AN, a core network for auditory processing, may show reduced connectivity with the SN, reflecting impaired perceptual sensitivity to salient auditory stimuli (Steiner et al. 2022). Similarly, the decreased connectivity between the AN and dSMN could affect auditory‐guided motor tasks, potentially due to long‐term hypoxia‐induced impairment of multisensory integration (Mallikarjun et al. 2018).

The reduced connectivity between the SN and dSMN suggests challenges in attention allocation and motor coordination, possibly due to the long‐term effects of hypoxia (Beheshtian et al. 2021). These changes may impair the ability to perceive and react in situations requiring rapid motor adjustments, such as emergency tasks, or affect motor planning in response to external stimuli (Li et al. 2024). Decreased connectivity between the pVN and both the dSMN and SN may indicate impairments in visual‐motor integration, potentially due to chronic anemia and hypoxia (Behroozmand et al. 2018). This could lead to difficulties in hand‐eye coordination and fine motor tasks requiring visual guidance (Iordan et al. 2024). Changes in DMN connectivity further suggest that chronic hypoxia may impair patients' ability to integrate internal and external cognitive functions, which could affect self‐reflection, emotional regulation, and motor coordination (Schimmelpfennig et al. 2023).

This study provides valuable insights into brain network changes in β‐TM patients, but several limitations must be addressed. First, the cross‐sectional design limits the ability to assess the dynamic processes of FC changes. Longitudinal studies are needed to better understand the long‐term relationship between these changes and clinical symptoms. Second, the absence of biomarkers such as iron metabolism or Hb concentrations prevents an exploration of how these factors influence brain network alterations. Future research should consider incorporating these physiological markers. Finally, while resting‐state fMRI data were presented, the study did not examine the relationship between task‐related brain activity and network changes. Combining task‐based fMRI studies, particularly those involving cognitive and emotional tasks, could provide a more comprehensive understanding. In addition, the lack of clinical scale analysis, including mood and cognitive assessments, prevents a direct correlation between observed brain connectivity changes and clinical symptoms. Incorporating these clinical measures in future studies could offer a more thorough understanding of neurofunctional changes in β‐TM patients and their progression over time.

In conclusion, our study provides comprehensive insights into the neural changes that occur in β‐TM patients, revealing significant alterations in both functional brain networks and inter‐network connectivity. These changes reflect both compensatory adaptive responses to chronic hypoxia and potential dysfunctions in higher‐order cognitive processes. This research suggests that long‐term hypoxia may lead to both compensatory mechanisms and disruptions in brain function, resulting in complex alterations in neural network interactions. The findings offer important implications for future research and potential interventions to mitigate the cognitive and emotional impairments experienced by β‐TM patients.

## Author Contributions

**Mingrui Yang**: Scan patients; Data analysis. **Peng Peng**: supervision. **Cheng Tang**: Scan patients. **Chaotian Luo**: Data anlysis. **Fei Peng**: Clinical data collection. **Rong Kong**: Data anlysis. **Chunxia Zhu**: Data preprocess. **Jiatong Liang**: Manage patients.

## Peer Review

The peer review history for this article is available at https://publons.com/publon/10.1002/brb3.70614

## Data Availability

The data that support the findings of this study are available from the corresponding author upon reasonable request.
